# Uptake of 18F-AV45 in the Putamen Provides Additional Insights into Alzheimer’s Disease beyond the Cortex

**DOI:** 10.3390/biom14020157

**Published:** 2024-01-29

**Authors:** Zhengshi Yang, Jefferson W. Kinney, Dietmar Cordes, The Alzheimer’s Disease Neuroimaging Initiative

**Affiliations:** 1 Cleveland Clinic Lou Ruvo Center for Brain Health, Las Vegas, NV 89106, USA; yangz@ccf.org; 2 Department of Brain Health, University of Nevada Las Vegas (UNLV), Las Vegas, NV 89154, USA; jefferson.kinney@unlv.edu; 3 Chambers-Grundy Center for Transformative Neuroscience, Pam Quirk Brain Health and Biomarker Laboratory, Department of Brain Health, School of Integrated Health Sciences, University of Nevada Las Vegas (UNLV), Las Vegas, NV 89154, USA; 4 Department of Psychology and Neuroscience, University of Colorado, Boulder, CO 80309, USA

**Keywords:** 18F-AV45 PET, putamen, striatum, Alzheimer’s disease, amyloid

## Abstract

Cortical uptake in brain amyloid positron emission tomography (PET) is increasingly used for the biological diagnosis of Alzheimer’s disease (AD); however, the clinical and biological relevance of the striatum beyond the cortex in amyloid PET scans remains unclear. A total of 513 amyloid-positive participants having 18F-AV45 amyloid PET scans available were included in the analysis. The associations between cognitive scores and striatal uptake were analyzed. The participants were categorized into three groups based on the residual from the linear fitting between 18F-AV45 uptake in the putamen and the cortex in the order of HighP > MidP > LowP group. We then examined the differences between these three groups in terms of clinical diagnosis, *APOE* genotype, CSF phosphorylated tau (ptau) concentration, hippocampal volume, entorhinal thickness, and cognitive decline rate to evaluate the additional insights provided by the putamen beyond the cortex. The 18F-AV45 uptake in the putamen was more strongly associated with ADAS-cog13 and MoCA scores (*p* < 0.001) compared to the uptake in the caudate nucleus. Despite comparable cortical uptakes, the HighP group had a two-fold higher risk of being ε4-homozygous or diagnosed with AD dementia compared to the LowP group. These three groups had significantly different CSF ptau concentration, hippocampal volume, entorhinal thickness, and cognitive decline rate. These findings suggest that the assessment of 18F-AV45 uptake in the putamen is of unique value for evaluating disease severity and predicting disease progression.

## 1. Introduction

Amyloid deposition in the cortex is well recognized as the defining characteristic of Alzheimer’s disease (AD) [1], which can be observed more than a decade before the onset of clinical syndromes [2]. Amyloid positron emission tomography (PET) imaging was established as a reliable technique to detect the brain amyloid status in vivo with high specificity [3,4,5]. Using the cortical uptake from amyloid PET scans to determine the brain amyloid status, by visual reading [3] or computational approaches [6,7,8], is increasingly used for participant enrollment in clinical trials and research. 

Based on the spatial extension of amyloid plaques observed in the post-mortem brain, multiple amyloid stages were defined with the amyloid spreading in the brain inferred as the temporal order of amyloidosis [9], which concluded that amyloid plaques exclusively accumulate in the cortex, particularly in the neocortex, in the initial stage and then spread to other regions in the later stages. The subcortex was demonstrated to be susceptible to amyloid pathology [9,10], but how strongly subcortical amyloid reflects clinical symptoms/progression and other pathological alterations remains to be examined. 

A few amyloid PET studies suggest additional clinical and biological benefits of including subcortical structures, particularly the striatum, for disease assessment compared to examining the cortex alone [11,12,13]. Patients in the high cortical and striatal uptake stage (stage 2) were found to have more severe tau pathology, smaller hippocampal volume, and steeper cognitive decline and hypometabolism compared to patients in the high cortical but low striatal uptake stage (stage 1) [11,12]. These studies suggest that the striatum may have unique value in predicting clinical syndromes and biological alterations beyond the cortex. However, while both stage 1 and stage 2 were categorized as high cortical uptake with the same lower bound, the average cortical uptake in stage 2 is substantially higher than in stage 1. Thus, the more rapid disease progression in stage 2 could be the collective influence of higher uptake in both the cortex and striatum, instead of being driven merely by the striatum. The extent to which the amyloid load in the striatum contributes to the differences observed between these stages remains unclear. 

To directly examine the additional insights provided by the striatum for AD assessment in amyloid PET scans requires a strategy to define groups/stages with comparable cortical uptake but distinct striatal uptake. In this way, we could isolate the contribution of the striatum from the cortex and then directly quantify its biological and clinical relevance in AD, including its association with clinical diagnosis, cognitive decline, genetic risks, and other pathological alterations. For example, it is well acknowledged that the *APOE* ε4 allele is the strongest genetic risk factor for sporadic AD, and the *APOE* genotype and cortical amyloid are confounding factors for each other [14]. The question is whether the ε4 allele leads to exacerbated subcortical amyloid burden between groups with comparable cortical amyloid load. In addition, evaluating if greater subcortical amyloid burden is associated with more rapid cognitive decline could help strengthen the clinical utility of amyloid PET in predicting disease progression. 

The putamen and caudate nucleus are the two major regions in the striatum, and they work closely in various neurological functions. Different parts of the striatum receive inputs from various cortical regions and then project their efferent outputs to the cortex through the thalamus [15]. Despite their close interactions, the caudate nucleus and putamen were observed to have differential roles in cognitive functions [16] and learning tasks [17]. These two regions play distinct roles in movement control, and their pathological alterations in parkinsonism and dyskinesia reflect distinct neuronal mechanisms [18], suggesting that they may experience different pathological alterations in a disease population. Therefore, it would be of interest to test if the putamen and caudate nucleus have differentiated susceptibility to brain amyloid pathology. 

In this study, we used 18F-AV45 PET data from a large cohort of participants in the AD continuum to investigate the biological and clinical relevance of amyloid deposits in the striatum, including both the caudate nucleus and putamen. Our analysis was primarily conducted on the putamen due to its striking association with cognition and larger group difference compared to the caudate nucleus. We hypothesized that the participants with higher putamen amyloid load are more likely to be ε4 carriers, are at a higher risk of being diagnosed with AD dementia, experience more severe pathological alterations, and suffer from faster cognitive decline.

## 2. Methods

### 2.1. Subjects

The data used in this study were obtained from The Alzheimer’s Disease Neuroimaging Initiative (ADNI, https://adni.loni.usc.edu/ (accessed on 8 February 2022)) database in February 2022. This study was approved by each participating ADNI site’s local Institutional Review Board as documented on the ADNI website. All participants gave written, informed consent. The sponsors for ADNI are listed in the Acknowledgements. Of the entire ADNI cohort, 1344 individuals were identified to have 18F-AV45 PET scans available. Because abnormal brain amyloid is the defining signature of AD and we aimed to assess the additional value provided by the striatum in the AD population, all participants included in the statistical analyses were required to be amyloid-positive based on 18F-AV45 PET scans to ensure participants in the AD continuum. The clinical diagnosis was based on the global clinical dementia rating score (global CDR) closest in date to PET scans as the diagnostic criterion for CN (global CDR = 0), MCI (global CDR = 0.5), and AD dementia (global CDR ≥ 1), together with the requirement of MMSE score for MCI/CN participants no less than 24/30 [11]. 

### 2.2. AV45-PET, MRI, and CSF Acquisition and Analysis

The PET data were acquired 50 to 70 minutes after a 370 MBq (10 mCi +/− 10%) bolus injection of radiotracer 18F-AV45. The PET images preprocessed through coregistration, averaging, standardizing image and voxel size, and smoothing to a uniform isotropic resolution were acquired from the database [6]. Each subject’s PET image was coregistered to the closest T1 structural MRI image. FreeSurfer 7.1.1 (https://surfer.nmr.mgh.harvard.edu/) was used to segment the T1 structural images. The original 18F-AV45 PET images were normalized by the mean intensity of the composite reference region [19,20] to derive the standardized uptake value ratio (SUVR). The cortical SUVR, frequently named the composite SUVR, was calculated by averaging over extensive regions consisting of frontal, anterior/posterior cingulate, lateral parietal, and lateral temporal regions [6]. An individual’s amyloid positivity was defined as the cortical SUVR of baseline PET scan greater than 0.826, determined from mixture modeling analysis [21]. The cutoff value was used as a subject inclusion criterion only to determine if a participant was in the AD continuum. Finally, the mean SUVRs in the putamen and caudate nucleus were computed. 

To evaluate if the partial volume effect on 18F-AV45 PET scans leads to underestimated radiotracer uptake in the striatum and compromises the statistical power to detect amyloid burden in the striatum, the PetSurfer processing pipeline (https://surfer.nmr.mgh.harvard.edu/fswiki/PetSurfer) was applied on 18F-AV45 PET scans to derive the striatal SUVR after partial volume correction (PVC). The default settings in PetSurfer were used, except that the default reference region Pons was replaced by the composite reference region, as mentioned above in the *mri_gtmpvc* command. It was found that the difference between amyloid-negative CN (CN-) and amyloid-positive participants was consolidated in the caudate nucleus but did not reach the difference as observed in the putamen without PVC (see Supplementary Figure S1). In contrast, PVC did not enhance but instead slightly deteriorated the difference between amyloid-negative CN (CN-) and amyloid-positive participants in the putamen. Therefore, the SUVRs computed without PVC are reported in the *Results* session. 

The hippocampal volume (sum of left and right hippocampus) was extracted from FreeSurfer output and then corrected for the total intracranial volume (TIV) to account for the differences in head size via a linear model: Vol_adj(i)_ = Vol_orig(i)_ – *b* × (TIV_(i)_ − Mean TIV), where *b* is the slope of hippocampal volume regressed on TIV, the mean TIV is the average TIV volume of all participants, and Vol_adj(i)_ and Vol_orig(i)_ are the corrected and original hippocampal volumes, respectively, for subject *i* [22]. The corrected hippocampal volume Vol_adj_ was used in the following statistical analysis. The average thicknesses of the left and right entorhinal cortex were also computed based on FreeSurfer output, which has the advantage of being insensitive to head size [23]. The CSF ptau concentration derived from the Elecsys ptau-181 immunoassay [24] was used to evaluate tau pathology, with the cut-off concentration for tau positivity determined to be 27 pg/mL [25]. The earliest visit with an AV45-PET scan available was treated as the baseline visit. The AV45-PET data, together with MRI data, CSF data, and neuropsychological tests from the baseline visits, were used in our cross-sectional analysis. 

### 2.3. Statistical Analysis

Considering that the full kinetic modeling validating 18F-AV45 was focused on the cortical regions with much less attention paid to the striatum, we first ran a two-sample t-test to examine if striatal SUVRs, similar to cortical SUVR, are elevated in amyloid-positive participants compared to CN- participants. After demonstrating the validity of striatal SUVRs, the remaining analysis was limited to amyloid-positive participants who are biologically determined to be in the AD continuum. 

A linear regression analysis was used to test the association of striatal SUVRs with age and cognitive scores, including both the 13-item Alzheimer’s Disease Assessment Scale-Cognitive Subscale (ADAS-cog13) and the Montreal Cognitive Assessment (MoCA). Because the putamen had stronger association with cognitive scores and larger group difference between CN- and amyloid-positive participants compared to the caudate nucleus (see Results), we exclusively focused on the putamen to quantify its additional value beyond the cortex for AD assessment in 18F-AV45 PET scan.

Among the amyloid-positive participants, a linear regression analysis between cortical SUVR (x) and putamen SUVR (y) was conducted with baseline PET data, y = intercept + *β* × x + residual r. The residual of an individual graphically is the signed distance of an individual away from the fitting curve (the residual is positive/negative for a subject located above/under the fitting curve), and it contains the remaining variance of putamen SUVR after accounting for cortical SUVR. 

As previously explained, the observed clinical and biological differences between amyloid stages can be the collective effect of both cortical and striatal SUVR in the amyloid staging approach [11]. To directly test the additional insights provided by the putamen, we categorized the participants into three groups based on the value of the residual r from the linear regression analysis between putamen SUVR and cortical SUVR. In detail, the residual r was first normalized by its estimated standard deviation to derive the standardized residual $r_{std}$. The participants having $r_{std}$ less than −0.5 were placed in the low putamen SUVR group (LowP), the participants having $r_{std}$ above 0.5 were grouped into the high putamen SUVR group (HighP), and the participants having $r_{std}$ in the range from −0.5 to 0.5 were placed into the middle putamen SUVR group (MidP). The cut-off value of 0.5 to separate these three groups was heuristically selected to ensure a relatively comparable number of participants in each group. An individual in the HighP group does not necessarily have a higher putamen SUVR than an individual in the LowP group, and vice versa. The group category depends on the 18F-AV45 uptake in the putamen and the cortex. Under the condition that participants have the same cortical SUVR, then an individual with a larger residual (e.g., HighP group) has stronger 18F-AV45 uptake or more severe amyloid burden in the putamen than an individual with a smaller residual (e.g., LowP group). 

The chi-square statistic was then used to evaluate whether the categorical measures, including clinical stages and *APOE* genotypes, were significantly different between LowP, MidP, and HighP groups. A one-way ANOVA followed by post-hoc 2-sample t-tests were used to examine the group differences of continuous measures, including hippocampal volume, entorhinal thickness, and CSF ptau concentration. A Bonferroni correction was used to adjust for the multiple comparisons. The corrected *p* value and Cohen’s d were reported. We then used a linear mixed effect (LME) model to evaluate the influence of putamen SUVR on cognitive decline rate with the formula ADAS-cog13~age at baseline + education + group category + years of follow-up × group category + (1|subject). ADAS-cog13 was used as the dependent variable for cognitive assessment, and a total of 2026 measures from baseline and follow-up visits were included in the analysis. Group category (LowP, MidP, and HighP), age at baseline visit, education, and years of follow-up were the fixed effects in the model. The intercept for each subject, namely (1|subject) was treated as the random effect in the model. The LME model was coded with full dummy variables; thus, no constant term was added to ensure the design matrix had full rank. The interaction term between years of follow-up and group category, namely years of follow-up * group category, was of particular interest. The β coefficients for the interaction terms represent the rate of cognitive decline for LowP, MidP, and HighP groups. MATLAB software (version 2023a, The MathWorks, Inc., Natick, MA, USA) was used for all statistical analyses. 

## 3. Results

### 3.1. Subject Characteristics

Based on the cortical SUVR, a total of 513 participants were determined to be in the AD continuum, namely amyloid-positive, including 67 CN participants, 334 participants with MCI, and 112 participants with dementia. The demographic characteristics of these participants are summarized in Table 1. A one-way ANOVA showed that years of education (*p* = 0.02), but not age (*p* > 0.05), was significantly different between clinical diagnoses. The chi-square test showed that gender (*p* = 0.0003) and *APOE* genotype (*p* = 0.03) were significantly different between clinical diagnoses. The percentages of female in CN, MCI, and dementia were 71.6%, 44.9%, and 49.1%, respectively. As expected, the MoCA and ADAS-cog13 scores were significantly different between these groups. 

**Figure 1:**
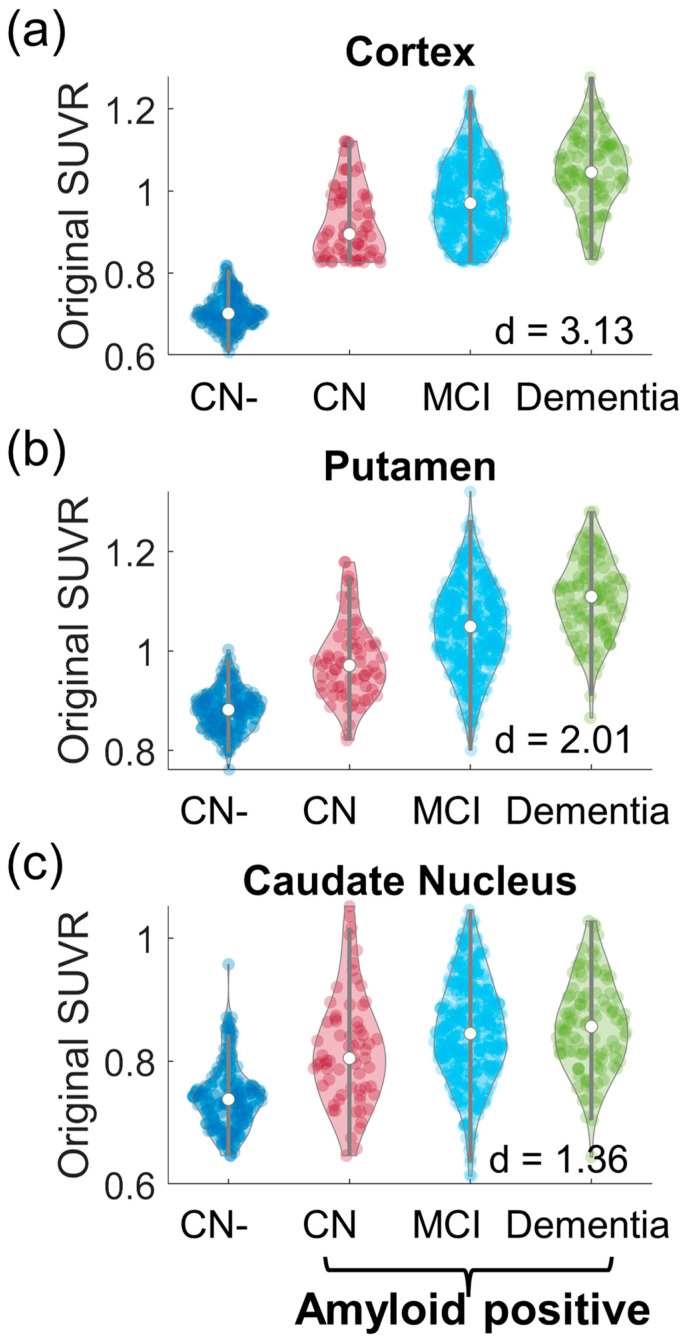
Violin plots of original SUVRs in the cortex (**a**), putamen (**b**), and caudate nucleus (**c**). A comparison between CN- and all amyloid-positive participants (including clinical diagnosis as CN, MCI, or dementia) was conducted with the Cohen’s d marked in the figure.

We first examined the differences in original striatal SUVRs between CN- participants (157 participants, mean (std) age 72.87 (7.2) years old) and all amyloid-positive participants to evaluate if the 18F-AV45 uptake in the striatum is evaluated in participants in the AD continuum. The violin plots of the SUVRs in the cortex, putamen, and caudate nucleus for CN- participants and the amyloid-positive participants with stratified clinical diagnosis are shown in Figure 1. Similarly to in the cortex (cohen’s d = 3.13), amyloid-positive participants had higher SUVRs in the putamen (cohen’s d = 2.01) and caudate nucleus (Cohen’s d = 1.55) than CN- participants, with the trend of elevated SUVR with disease progression. 

### 3.2. SUVRs in the Putamen and Caudate Nucleus Have Differentiated Association with Cognitive Scores and Age

**Figure 2:**
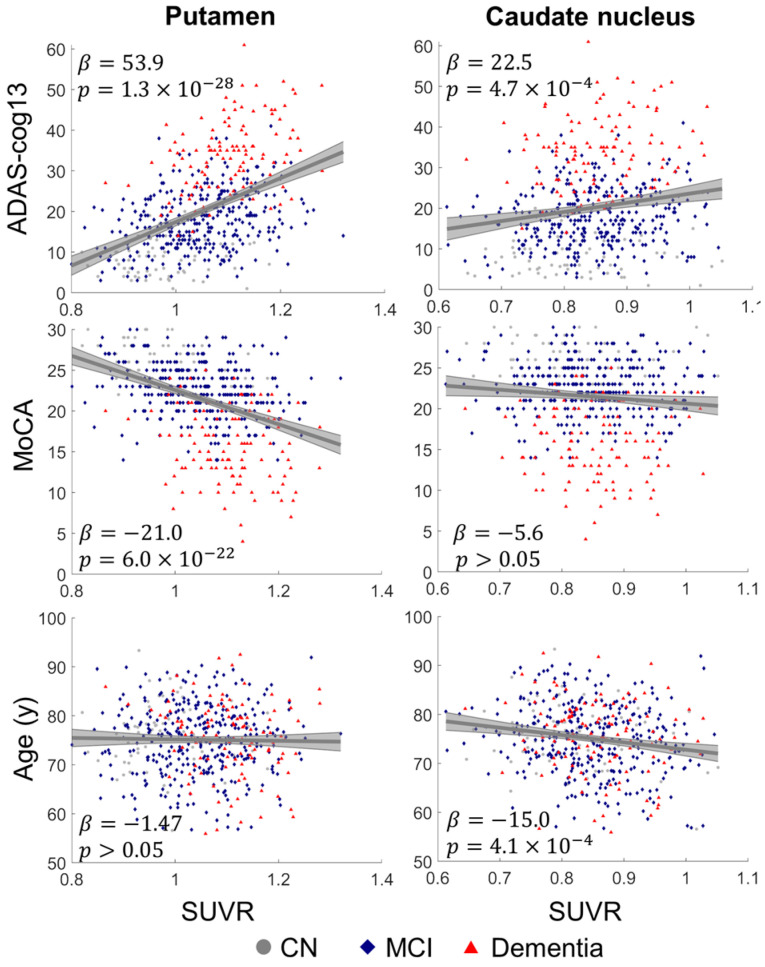
Association analysis between striatal SUVRs and cognitive scores/age. ADAS-cog13 and MoCA were used for cognitive assessment. All amyloid-positive participants, including CN (gray dot), MCI (blue diamond), and dementia (red triangle), were used for analysis. The linear fitting curves with 95% confidence interval (gray areas), together with scatter plots, were shown in the figure. The slopes β from the linear fitting and the corresponding significance level are marked in the figure. The putamen had stronger association with cognitive scores, and the caudate nucleus had stronger association with age (*p* < 0.05).

The scatter plots between cognitive scores/age and striatal SUVRs, together with the linear fitting curve with 95% confidence interval, are shown in Figure 2. The ADAS-cog13 was associated with SUVR in the putamen (β = 53.9, Bonferroni-corrected *p* = 1.3 × 10^−28^) and caudate nucleus (β = 22.5, *p* = 4.7 × 10^−4^). MoCA score was associated with SUVR in the putamen (β = −21.0, *p* = 6.0 × 10^−22^) but not in the caudate nucleus (β = −5.6, *p* > 0.05). The putamen had stronger associations than the caudate nucleus for both ADAS-cog13 (student’s *t* = 4.37, *p* = 6.9 × 10^−6^) and MoCA scores (student’s *t* = −4.76, *p* = 1.1 × 10^−6^) based on Student’s *t* statistic. The cortical SUVR had similar association with cognitive scores as the SUVR in the putamen but with weaker strength (Supplementary Figure S2).

Age was found to be negatively associated with SUVR in the caudate nucleus (β = −15.0, *p* = 4.1 × 10^−4^) and not associated with SUVR in the putamen (β = −1.47, *p* > 0.05). The caudate nucleus had significantly stronger association with age than the putamen (student’s *t* = 2.69, *p* = 0.0036). Collectively, the putamen had 18F-AV45 uptake more closely associated with cognitive impairment and was less sensitive to the age effect than the caudate nucleus. In addition, the putamen had a larger SUVR difference between CN- and amyloid-positive participants compared to the caudate nucleus. Therefore, the remaining analysis was limited to the putamen, and we focused on evaluating the additional value of assessing putamen amyloid beyond cortical amyloid in terms of its clinical, biological, and genetic relevance in AD. 

### 3.3. Group Categorization

**Figure 3:**
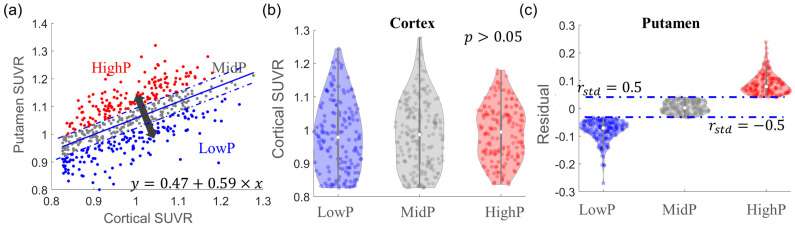
Participant categorization based on the association of SUVRs in the cortex and putamen. (**a**) Scatter plot between cortical SUVR and putamen SUVR. The linear fitting curve (solid blue line) is also shown in the figure. Based on the standardized residual (*r_std_*) from the linear fitting, the participants were grouped into LowP (*r_std_* ≤ −0.5; blue), MidP (|*r_std_*| < 0.5; gray), or HighP (*r_std_* ≥ 0.5; red) groups along the direction (black arrow) perpendicular to the fitting curve. (**b**) Violin plots of original cortical SUVR in LowP, MidP, and HighP groups. There was no difference in cortical SUVR between these three groups (*p* > 0.05). (**c**) Violin plots of the residual from the linear fitting. The dashed blue lines in (**a**,**c**) indicate *r_std_* = ±0.5.

The linear fitting curve between cortical SUVR (x) and putamen SUVR (y) was estimated to be y = 0.47 + 0.59 × x, as shown in Figure 3a. Cortical SUVR explained 39% variance (namely R^2^ = 0.39) in SUVR in the putamen; meanwhile, 61% variance in SUVR in the putamen, namely the residual, was not explained by the cortex, which was significantly associated with ADAS-cog13 and MoCA scores (*p* < 0.05, not shown in figure). The participants were then categorized into three groups based on the residual with the magnitude of the order of LowP (157 participants; blue dots) < MidP (200 participants; gray dots) < HighP (156 participants; red dots) groups, as shown in Figure 3c. The cortical SUVR in these three groups (violin plots in Figure 3b) was not significantly different between groups (*p* > 0.05 in one-way ANOVA test). 

### 3.4. Clinical and Biological Differences between LowP, MidP, and HighP Groups

**Figure 4:**
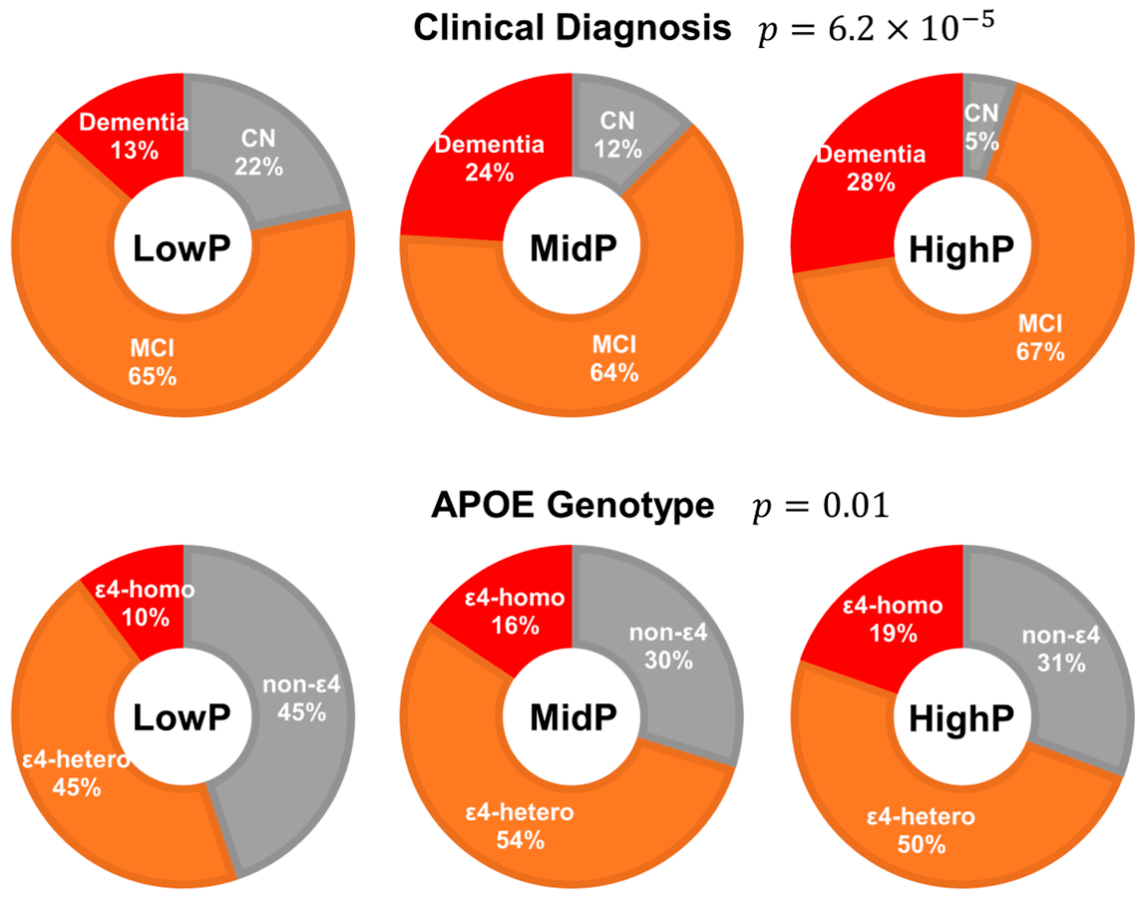
Proportions of clinical diagnosis (**top panel**) and *APOE* genotype (**bottom panel**) in LowP, MidP, and HighP groups. Clinical diagnosis and *APOE* genotypes are significantly different in LowP, MidP, and HighP groups.

The chi-square tests show that the proportions of clinical diagnosis (*p* = 6.2 × 10^−5^) or *APOE* genotype (*p* = 0.01) were significantly different between the LowP, MidP, and HighP groups (see Figure 4). The percentage of participants with dementia increased from 13% in the LowP group to 28% in the HighP group, and the percentage of CN participants decreased from 22% in the LowP group to only 5% in the HighP group, with approximately 65% of MCI in all groups. In terms of *APOE* genotype, participants in HighP group were more likely to be ε4-homozygous (19% in the HighP group and 10% in the LowP group), and the participants in MidP and HighP groups were less likely to be non-ε4 carriers (45% in LowP, 30% in MidP, and 31% in HighP). 

**Figure 5:**
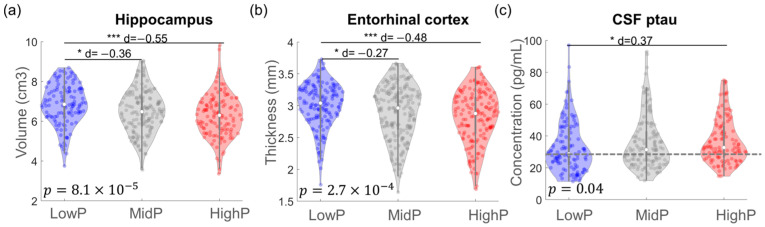
Group comparisons of hippocampal volume (**a**), entorhinal thickness (**b**), and CSF ptau concentration (**c**) between LowP, MidP, and HighP groups. One-way ANOVA found that these measures were significantly different between three groups. The horizontal line in (**c**) indicates the cut-off concentration for tau positivity. The Cohen’s d values for the pairwise comparisons having significant differences were marked in the figure. * *p* < 0.01, *** *p* < 1 × 10^−5^.

The LowP, MidP, and HighP groups had significantly different hippocampal volume (*p* = 8.1 × 10^−5^), entorhinal thickness (*p* = 2.7 × 10^−4^), and CSF ptau concentrations (*p* = 0.04) as shown in Figure 5. For the LowP, MidP, and HighP groups, the mean ± standard deviation hippocampal volumes were 6.85 ± 1.02, 6.47 ± 1.07, and 6.27 ± 1.07 cm^3^, respectively; the mean ± standard deviation entorhinal thicknesses were 3.01 ± 0.35, 2.90 ± 0.43, and 2.82 ± 0.41 mm, respectively; and the mean ± standard deviation CSF ptau concentrations were 33.0 ± 15.9, 35.9 ± 16.7, and 37.3 ± 14.4 pg/mL, respectively. The HighP group had the smallest hippocampal volume, thinnest entorhinal thickness, and highest CSF ptau concentration compared to the other groups. The effect sizes are marked in the figure for the pairwise t-tests showing significant group differences. 

### 3.5. Association of Putamen Amyloid with Cognitive Decline

The beta coefficients estimated from the LME model are shown in Table 2. The ADAS-cog13 score was not associated with the age at baseline (β = 0.02, *p* > 0.05). Higher education was associated with better cognition, namely lower ADAS-cog13 score (β = −0.65, *p* = 0.001). The β coefficients for LowP, MidP, and HighP were 25.5, 29.9, and 31.9, respectively. The interaction terms between group category (LowP, MidP, and HighP) and year of follow-up were significantly associated with ADAS-cog13 score, with the coefficients as 1.12, 1.90, and 2.85 for the LowP, MidP, and HighP group, respectively, where a larger coefficient indicates more rapid cognitive decline. 

## 4. Discussion

In this study, we assessed the additional value of the striatum beyond the cortex in the 18F-AV45 amyloid PET scans among a large cohort of participants in the AD continuum. With few studies reporting the value of striatum beyond the cortex in amyloid PET scans, we demonstrated the discrepancy between the putamen and the caudate nucleus. More importantly, we used a novel approach to quantify the additional insights provided by striatal amyloid in evaluating disease severity and predicting cognitive decline. 

Although the putamen and caudate nucleus are structurally and functionally close to each other, the 18F-AV45 uptake in the putamen had better discriminative power in differentiating amyloid-positive participants from CN- participants. The SUVR in the putamen had a significantly stronger association with cognitive assessment and a weaker association with age compared to the SUVR in the caudate nucleus. This finding suggests that the 18F-AV45 SUVR in the putamen can be a more robust imaging marker reflecting AD disease progression than that in the caudate nucleus. Likely because of the weak association in the caudate nucleus, striatal amyloid (combining the putamen and the caudate nucleus) was previously found to have a marginal, non-significant correlation with cognition [11]. Age was found to be negatively associated with the SUVR in the caudate nucleus; such an association was not observed in the putamen. The negative association in the caudate nucleus could be due to the partial volume effect in PET scan or brain atrophy, which could lead to underestimated 18F-AV45 uptake. Such an explanation is supported by the observation that the caudate nucleus had consolidated group difference between CN- and amyloid-positive participants after PVC. The differentiated statistical power for group comparison and association analysis suggests the necessity to evaluate 18F-AV45 uptake in the putamen separate from the caudate nucleus. Note that amyloid burden in the striatum is not unique in sporadic AD, which is also observed in familial AD cases [26,27]. Unlike sporadic AD cases, as investigated in this study, having elevated striatal amyloid deposition at the later stage, patients with familial AD have amyloid aggregated in the striatum since the initial stage of amyloidosis [26,27]. Therefore, cautions need to be taken when generalizing the findings from this study to the familial AD population. 

A large fraction of the variance of the SUVR in the putamen could not be explained by cortical SUVR. The SUVR in the putamen consistently showed a significant association with cognition. Such an association was still observed with the SUVR in the putamen after adjusting for cortical SUVR, namely the residual from the linear fitting. By categorizing participants into three groups with strictly separated residual but comparable cortical SUVR, the participants in the HighP group had twofold likelihood to be diagnosed with dementia and only one quarter chance to be diagnosed with CN compared to the LowP group, suggesting the close relevance of putamen amyloid with the clinical manifestation. In addition, although participants in all groups had comparable cortical amyloid burden, the participants in the MidP and HighP groups were more likely to be ε4-homozygous and less likely to be non-ε4 carriers compared to the LowP group. The existence of the ε4 allele was well observed to be associated with higher amyloid burden in the cortex [14]. Our study suggests that, among participants with comparable cortical amyloid load, the ε4 allele might make the putamen more susceptible to amyloid pathology. Furthermore, the participants with high putamen amyloid had smaller hippocampal volume, thinner entorhinal thickness, and CSF ptau concentration than the participants with lower putamen amyloid. These findings support that putamen amyloid is not a proximity of cortical amyloid but has its own value in assessing AD disease progression. Evaluating the putamen amyloid, particularly alongside cortical amyloid, is beneficial for AD assessment, which is consistent with previous striatal amyloid PET staging studies [11,12]. Differently from these studies combining the putamen and caudate nucleus for assessment and having a mixed effect driven by both cortical and striatal amyloid between stages, our study demonstrates the necessity to analyze the 18F-AV45 uptake in the putamen and caudate nucleus separately. Furthermore, higher putamen amyloid was associated with more rapid cognitive decline in the LME model, where the HighP group had more than twofold faster cognitive decline than the LowP group based on the estimated coefficient. These findings are in line with our hypothesis that, despite the same level of cortical amyloid burden, an individual with higher putamen amyloid is more likely to experience more severe clinical syndromes and pathological changes. Previous reports found that a notable fraction of participants with abnormal amyloid PET (abnormality was determined based on cortical regions) did not show cognitive deficits [28,29], possibly because these subjects continued to have low putamen amyloid (e.g., LowP group) despite elevated cortical amyloid. Collectively, these results indicate that, from both clinical and biological perspectives, 18F-AV45 uptake in the putamen provides valuable insights of an individual’s vulnerability to AD pathology. The categorization of LowP, MidP, or HighP groups could be a potential approach to differentiate if an individual is under slow or rapid disease progression toward dementia. 

There are a few limitations in the study. First, the biological interpretation of AV45 SUVR in the striatum was not extensively examined. The full kinetic modeling of AV45-PET scan was focused on the cortical and reference regions, such as the cerebellum and white matter [30], while little attention was paid to the striatal regions. With the available dataset, we could not conduct the kinetic analyses. However, the striatal uptake showed a similar trend to the cortical AV45 uptake between CN- and amyloid-positive participants or between different clinical diagnoses among amyloid-positive participants, suggesting that the striatal SUVR likely reflects the amyloid burden in the striatum instead of being driven by non-specific binding. Second, both the medial temporal lobe and striatal-frontal network are the two fundamental neural systems supporting episodic memory and executive function [31,32]. A functional MRI study revealed that the functional demand from the hippocampus in a virtual navigation task could shift toward striatal regions during the aging process [33], suggesting the interplay between the medial temporal lobe and striatum. It would be of interest to test the potential causal effect of putamen amyloid on hippocampal atrophy at the individual level, which may help develop therapeutic strategies to delay or prevent the neurodegenerative process. Third, the current study is limited to amyloid-positive participants without considering the amyloid-negative participants, who might be experiencing amyloidosis but have not yet reached the pathological level. Because amyloid-negative participants are not destined to be in the AD continuum, they were not included in the analysis. In addition, considering that amyloidosis occurs in the cortex prior to the striatum based on the amyloid staging, it is less likely that striatal amyloid has a substantial effect before cortical amyloid reaching the pathological level. Fourth, AD dementia is characterized by declines in various cognitive domains, such as learning and memory, language, executive function, complex attention, perceptual-motor, and social cognition [34]. Using domain-specific cognitive assessment and functional MRI data could be beneficial to clarify which brain functional networks are disrupted particularly relevantly to amyloid accumulation in the putamen, especially considering the multi-faceted roles of the striatum in various functionally segregated circuits [35]. Future studies are warranted to clarify the relevance of elevated putamen amyloid with brain function. 

In summary, the 18F-AV45 uptake in the putamen is closely related to disease severity and other AD pathological hallmarks, which is also predictive of the future cognitive decline rate. The *APOE* ε4 allele might make the putamen more susceptible to amyloid pathology. These findings suggest that amyloid burden in the putamen could have a unique value in evaluating disease severity and predicting disease progression. The assessment of amyloid pathology in the putamen might be critical to understanding the pathological mechanisms in AD and thus aid in the development of more effective therapeutic strategies. 

## Figures and Tables

**Table 1:** Demographic characteristics of the participants enrolled in the study.

	CN	MCI	Dementia	*p* Value
Diagnosis	67	334	112	
Age	75.9 (6.3)	74.9 (7.1)	75.4 (7.5)	0.43
Education (years)	16.4 (2.6)	16.0 (2.7)	15.3 (2.6)	0.02
Female sex, no. (%)	48 (71.6)	150 (44.9)	55 (49.1)	0.0003
MoCA	25.9 (2.2)	22.6 (3.1)	15.6 (4.5)	8.2 × 10^−49^
ADAS-cog13	8.5 (4.5)	17.8 (7.0)	34.0 (8.9)	7.8 × 10^−58^
*APOE* status, non-ε4/ε4-hetero/ε4-homo *	32/31/4	113/164/52	31/59/21	0.03

* non-ε4: no ε4 allele; ε4-hetero: ε4 heterozygous carriers with one ε4 allele; ε4-homo: ε4 homozygous carriers with two ε4 alleles.

**Table 2:** The β coefficients from linear mixed effect model for assessing the association of putamen amyloid with cognitive decline.

Fixed Effect	Beta	SE	*t* Value	*p* Value
Age	0.02	0.08	0.26	0.80
Education	−0.65	0.20	−3.18	0.0015
LowP	25.49	6.95	3.67	0.0003
MidP	29.87	6.88	4.34	1.5 × 10^−5^
HighP	31.91	6.99	4.56	5.3 × 10^−6^
LowP × years of follow-up	1.12	0.13	8.94	8.9 × 10^−19^
MidP × years of follow-up	1.90	0.12	15.23	1.2 × 10^−49^
HighP × years of follow-up	2.85	0.15	18.59	2.6 × 10^−71^

## Data Availability

The dataset supporting the conclusions of this article is publicly available in The Alzheimer’s Disease Neuroimaging Initiative (ADNI) database (http://adni.loni.usc.edu/).

## Supplementary Materials

The following supporting information can be downloaded at: https://www.mdpi.com/article/10.3390/biom14020157/s1, Figure S1: Violin plots of SUVRs after partial volume correction in the cortex (a), putamen (b), and caudate nucleus (c). A comparison between CN- and all amyloid-positive participants (including clinical diagnosis as CN, MCI, or dementia) was conducted with the Cohen’s d marked in the figure. Figure S2: Association analysis between striatal/cortical SUVRs and cognitive scores/age. ADAS-cog13 and MoCA were used for cognitive assessment. All amyloid-positive participants, including CN (gray dot), MCI (blue diamond), and dementia (red triangle), were used for analysis. The linear fitting curves with 95% confidence interval (gray areas), together with scatter plots, were shown in the figure. The slopes β from the linear fitting and the corresponding significance level were marked in the figure. The putamen had stronger association with cognitive scores, and the caudate nucleus had stronger association with age (*p* < 0.05).
