# Supplementary material for: Uptake of 18F-AV45 in the Putamen Provides Additional Insights into Alzheimer’s Disease beyond the Cortex
Source: Biomolecules. 2024 Jan 29;14(2):157. doi: 10.3390/biom14020157 (PMC10886857; doi:10.3390/biom14020157)
Supplement: Supplementary file 1 [file biomolecules-14-00157-s001.zip › biomolecules-2746576-supplementary.pdf]

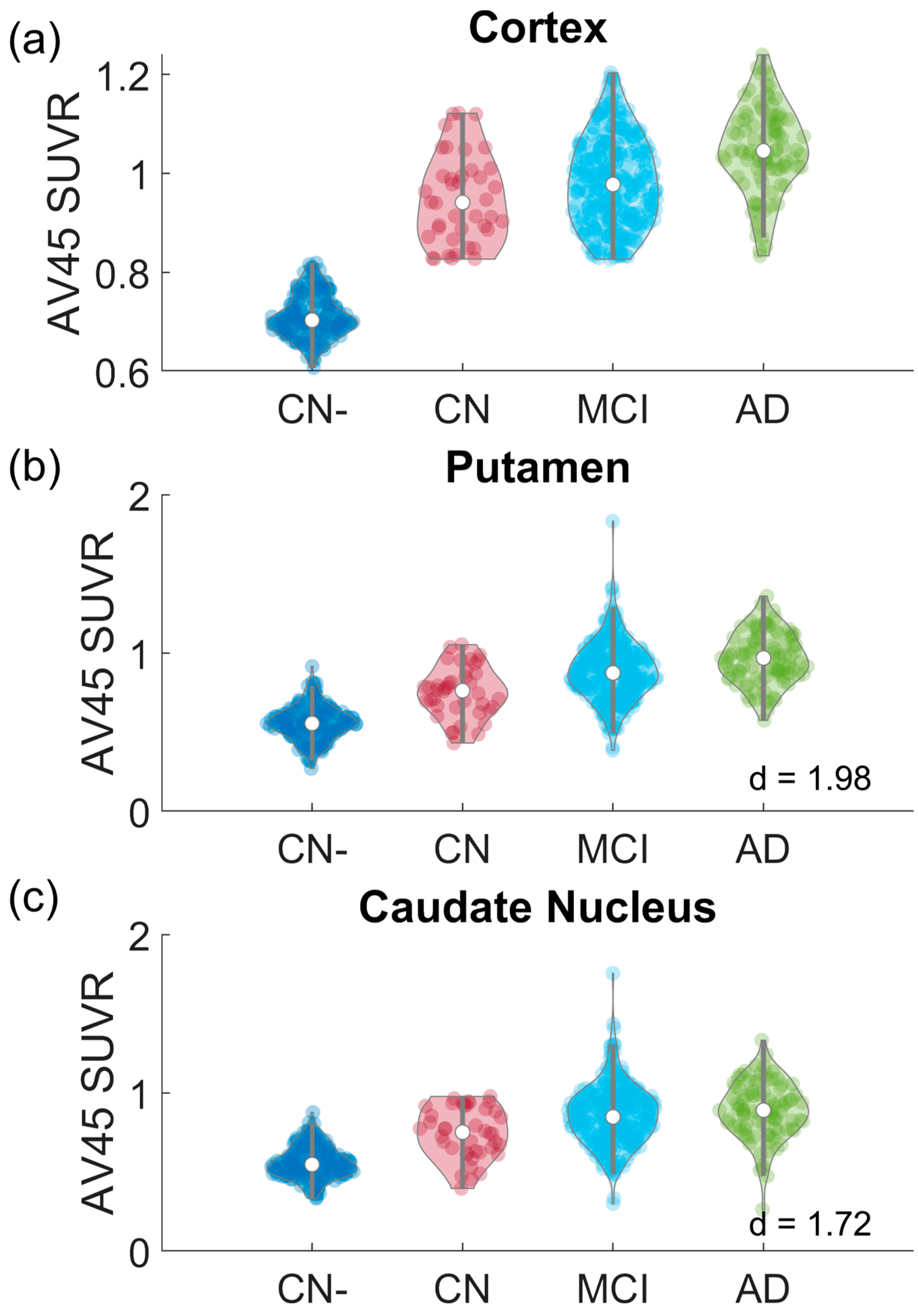

Figure S1. Violin plots of SUVRs after partial volume correction in the cortex (a), putamen (b), and caudate nucleus (c). A comparison between CN- and all amyloid-positive participants (including clinical diagnosis as CN, MCI, or dementia) was conducted with the Cohen's d marked in the figure.

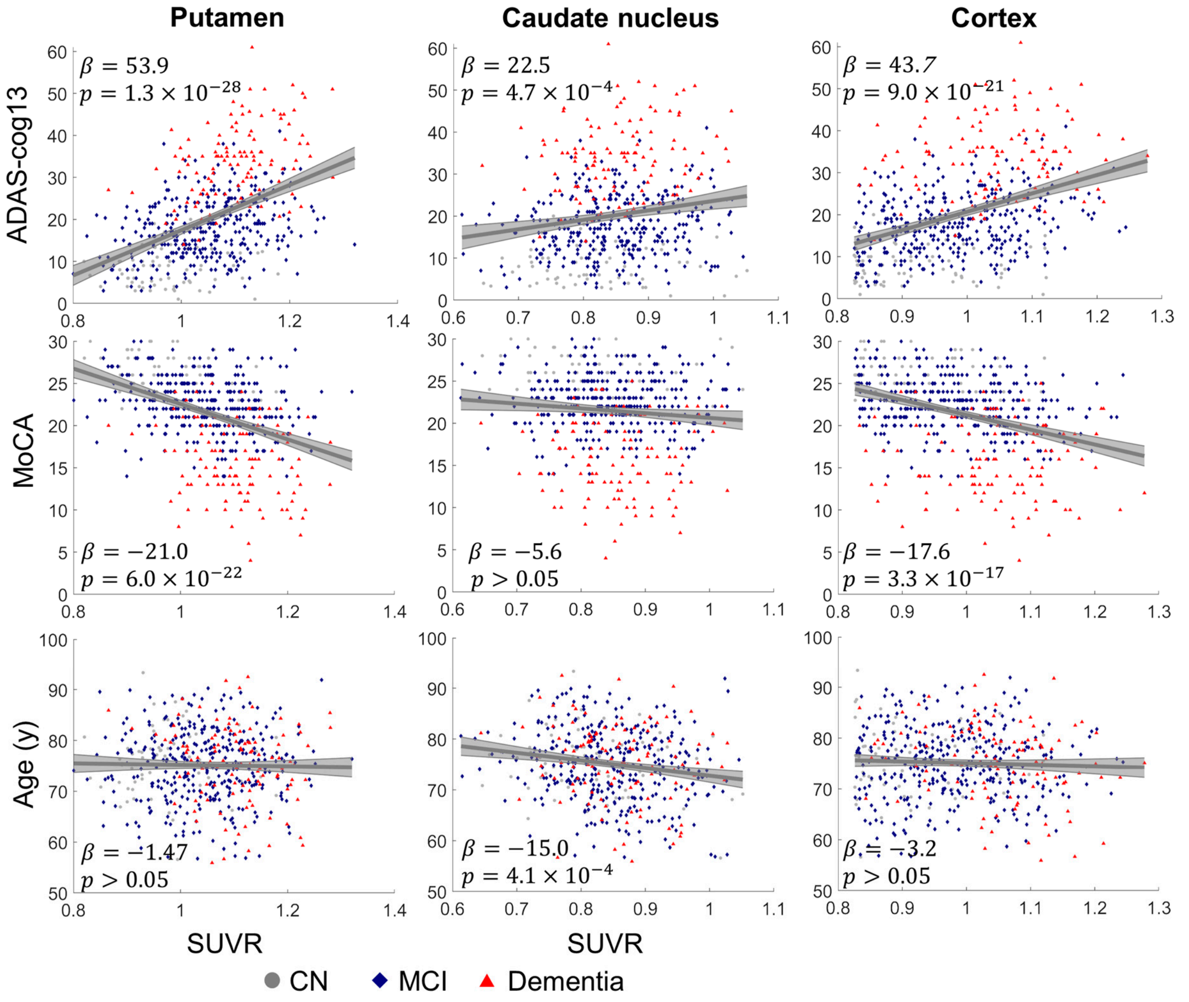

Figure S2. Association analysis between striatal/cortical SUVRs and cognitive scores/age. ADAS-cog13 and MoCA were used for cognitive assessment. All amyloid-positive participants, including CN (gray dot), MCI (blue diamond), and dementia (red triangle), were used for analysis. The linear fitting curves with 95% confidence interval (gray areas), together with scatter plots, were shown in the figure. The slopes β from the linear fitting and the corresponding significance level were marked in the figure. The putamen had stronger association with cognitive scores, and the caudate nucleus had stronger association with age ($p < 0.05$).
